# Fibrinous Bodies in the Heart after Death

**Published:** 1856-02

**Authors:** Henry Cady

**Affiliations:** Monson, Mass.


### *Fibrinous Bodies found in the Heart after Death.—Report of three cases.*

By HENRY CADY, M. D., Monson, Mass.

(Communicated for the Boston Medical and Surgical Journal.)

CASE I.—This case occurred in February, 1847. The subject was a young man aged 20 years, operative in a Woollen Mill, not very robust, of rather lax fibre and phlegmatic temperament. He had never been the subject of much disease requiring medical interference until the above date, at which time (Feb. 11, 1847) he was attacked with pleuro-pneumonia, embracing mainly, at the outset, the lower half of the right lung, but in its progress extending to, and involving the left. The disease was somewhat severe, but yielded to the lancet and other antiphlogistic treatment, so that by the ninth day from the attack, the patient had become comfortable. The respiration was comparatively easy; the matter expectorated was yellowish, free from any blood, and easily thrown off without much coughing. All these favorable indications continued and increased during the two following days. On the twelfth day of the disease I visited the patient at 11 o'clock, A. M. He had rested well the previous night; respiration and expectoration easy, tongue moist and almost clean, skin soft and of natural temperament, pulse 75 per minute, soft and uniform; no pain; begs for something to eat, "wants something better than weak broth and gruel." The medicines at this time employed, as well as on the previous day, were syr. serpentar., senega, and liquorice, and occasionally a Dover's powder and mucilages. My directions were to continue as on the day previous. About a quarter past 11 o'clock, I left the house, and before noon the young man was *dead !*

It may be readily inferred that I was utterly confounded when, on my return home in the evening, the fact of his death was told me. The mother of the young man (a widow) made to me the following statement :—"You had been gone but a little while; I was out in the cook-room preparing for dinner, when I heard from the sick room an unusual noise, like choking or calling, or something of the sort; I thought that perhaps he was sick at the stomach. I went immediately into the room and found him stretched upon his back, head tipped backwards, eyes rolled upwards, and he was struggling for breath, but could not breathe at all, seemingly; the arms were stiff (convulsed,) I didn't know but he was dying, but thought at first that it *could not be so.* I ran and called Mrs. ——, (a woman in an adjoining tenement,) and when I returned he scarcely stirred or gasped. He was dead.

*Post-mortem* examination, twenty hours after death. The body, externally, showed nothing worthy of note. The examination was carried no father than to observe what pertained to the thoracic and abdominal viscera, the latter of which showed no marks of disease. On opening the thorax, the lower half of the membrane lining the ribs, and a like portion reflected over the lower half of both lungs, fully exhibited the "footprints" of disease. At two or three points there was slight adhesion, very easily severed. From three to six ounces of effused serum were found in the sac of the pleura—more on the left than on the right side. The vessels of the membrane were considerably congested, and of a dull-red color. These marks were stronger on the left side than on the right, thus corresponding well with the fact, that after the acute stage of the disease had passed by, this point (the left) was the last to yield. The last blister was applied to the left side. The whole appearance, in fine, of both the substance of the lungs and of the investing membrane, was such as might be expected in the first stage of recovery from inflammatory disease–such, for instance, as is apparent in a similar condition of the sclerotic coat of the eye.

The external appearance of the heart was natural; there was a teaspoonful or two of serum in the pericardium. On opening the cavities, no structural lesion was apparent; but in the left ventricle was found a body of rather flattened shape, about three inches long, rather more than three eighths of an inch wide at its central portion, growing narrower towards each extremity, and a little more than an eighth (nearly one fourth (of an inch thick. The color was pale-red, and in solidity and firmness equal to the stem of a mushroom. It resembled in shape a large-sized domestic leech, as this animal appears when leisurely moving in water, (the motion, of course, excepted.) One end of this body had a very slender attachment—hardly more firm than would be sufficient to sustain its own weight —to about the middle of the left wall of the ventricular cavity; the other end was pushed through into the aorta, and was closely embraced by the semilunar valves.

CASE II.—This was a female child that died at the age of five months and thirteen days. Early in February, 1852, up to which date the child had always been healthy, it had an attack of "lung fever." I saw the child but once during the course of this disease. A good old lady in the neighborhood, partial to homœopathy, and who kept a little wallet of infinitesimals for the good of her neighbors, prescribed for the patient, and her remedies, together with some domestic medicines employed by the mother, constituted the treatment for about a week from the onset of the disease. The good doctress having herself become ill, was unable to attend farther to her charge, and at this stage of the disease I was called, *once*, and accordingly prescribed and left medicine for the child. I was informed by the mother that it had been "very sick," that the fever had been "very high," the air-passages had been badly "choked with phlegm," that there had been "hard cough with panting, wheezing and rattling in the chest." The child still had general fever at the time I saw it, though of moderate intensity. The mother said it was not "nearly so high as it *had* been." There was a wheezing sound through the air-passages in the lungs, more apparent in the left than in the right bronchi. I prescribed an emetic of ipecac., to be followed, at what I judged to be suitable intervals, by hive-syrup, liquorice water, and a free use of mucilages. I saw the patient no more till nearly three weeks from the time of the above-mentioned visit. At this period, hav ng been again called, the mother stated to me that the fever and disease of the lungs passed off within a few days after my former visit, that the child had *now* recovered, so that it appeared "most of the time as well as ever," but for the last two weeks it had been subject to sudden attacks of "something that made it appear as though it was choked;" that she had repeatedly passed her finger into its mouth, and down its throat, believing there *must* be something there choking it. These "spells," she said, for the first few days did not last more than half a minute, but came on every day, and on some days two or three times. She was alarmed ; because the child, while the "spells" lasted, seemed greatly distressed, the hands and feet were thrust upwards; face flushed, sometimes of a dark crimson color ; there was intense struggling for breath and an appearance as "though the child would choke to death ;" still, as soon as the paroxysim has passed by, it resumed its wonted appearance, took its food (from the breast) with good relish and in accustomed quantity ; and it appeared as though nothing had been the matter with it."

These spells, according to the information given by the mother, always came on while the child was lying quietly on its back, either in the cradle or on the lap, and never while in a sitting postuie, or when lying on either side. For the last five or six days preceding this visit, the mother informed me that the paroxysms had lasted longer than formerly, and that in addition, the child had, at each attack, been convulsed throughout its whole body. The night preceding the morning I was called to the child (this second visit,) it had had two "fits," more severe and lasting longer than any preceding. These two were judged to have lasted five minutes or more, each. On the morning of the visit, however, nothing of actual disease could be detected in the case. The child was lively and playful ; pulse, skin, tongue, &c , natural ; the gums were not swollen or red, but appeared as at the very first indications of approaching teeth. Judging that there might be some derangement of the first passages, either primary or by reason of some glandular defect, I prescribed a small laxative and alterative dose of rhubarb and hydrag. cum. creta, and advised the use, for a few days, of assafœtida and fluid ext. valerian. Whether or not any condition which followed was attributable to the means employed, I will not venture to say, but for nearly two weeks subsequently, there was an almost entire absence of the "fits," and the child appeared to be well. Notwithstanding, at the expiration of the two weeks, the "trouble" again returned and soon became more formidable than ever. A "fit" came on at this time, every day, for five or six days in succession, and on two of these days, as nearly as can be recollected, there were two additional fits, each successive one assuming a more grave character than the former. A variety of treatment was employed; the warm bath, anti-spasmodics and alteratives were resorted to, and at the suggestion of counsel, the gums were freely cut, though seemingly a forestalling of treatment. At the end of the foregoing period, an interval of four or five days followed, in which the child was exempt from any convulsions, and appeared quite bright and natural again. Nevertheless, at the close of the last period, a paroxysm more severe than any that had yet arisen, occurred, lasting more than half an hour. The evening following—this last having taken place in the morning—the child was seized with a succession of epileptic convulsions, with very short intervals, and sometimes scarcely any, till the next morning at daybreak, when it became still and quiet in death. The *post-mortem* examination took place twelve hours after death. It may be sufficient to state here, that no morbid appearances were detected in the case. Close inspection was made of all the contents of the thorax and abdomen—the brain and spinal cord were *not* examined. The left ventricle of the heart contained a small body about two inches in length; in shape somewhat more cylindrical than the one described in Case I.; in thickness equal to a common-sized goose quill, moderately flattened. In all its essential properties, it bore such a striking resemblance to that of Case I. that it is deemed uncourteous to ask for room in the choice pages of a Medical Journal for an extended detail. One end of the body stuck to the wall of the ventricle, though with *hardly* any, if *any* stronger tenure, than might be expected from mere attraction; the other was lodged between the semilunar valves, but did not extend through into the aorta.

Case III.—This was a male child, nearly two years of age (1 year, 8 months and 24 days.) The child had been healthy from its birth till the middle of last September (1855.) During the latter half of the previous summer it was undergoing the last part of the process of teething, and had for some weeks more or less diarrhœa, but never so profuse as to make it sick, or cause any more than a small degree of emaciation. Nothing but simple domestic remedies were used, or required for this (so common) condition. The teeth were all through, the gums healthy, and diarrhœa had subsided, at the time of its seizure by the disease to be described hereafter. The middle of last September, this child was attacked with dysentery; attended, for the first few days, with but slight general fever, though it afterwards became augmented, so that for a week following, it was fully of medium intensity. The stools were not very frequent, not repeated oftener than from two to four hours more or less bloody and mucous during the time of highest general fever; tenesmus moderate. Further description is needless. The case in no respect differed from ordinary cases of dysentery of medium severity. At the end of two weeks from the onset of the disease, the child was convalescent and appeared to be doing well; the appetite had returned, the stools were no more frequent than from two to four in twenty-four hours, marked still with some mucous and watery matter, but uniformly progressing towards a natural state. At this period the child was "getting about" and nearly free from manifestations of suffering. The medicines at this time employed were a syrup prepared from the Lima bark, with the addition of a little brandy every eight hours, with a Dover's powder at bedtime. After a continuation of these favorable indications for four or five days, one night, after midnight, the child, having rested quietly till then, was seized suddenly with a severe convulsion, which, in spite of remedies, warm bath included, lasted more than four hours. Twelve hours afterwards, however, it appeared nearly as well as before the fit. The night following it had a slight paroxysm, lasting about five minutes; and on the night still succeeding, one slighter still, lasting no more than half a minute. There were no more appearances of convulsive action, till nearly the close of the third day from the last-named period, when it was seized, near sunset, with a fit more severe than the first, and continued profoundly convulsed, in spite of every effort made to relieve it, for nearly twelve hours, when death closed the scene.

The *post-mortem* examination was commenced twelve hours after death. The viscera of the abdomen were examined, when, night coming on, the brain and thoracic viscera were left to be examined on the following morning. The mucous lining of the colon, throughout, showed the marks of disease; patches were found scattered along its course, still retaining a faint red hue. Other portions had nearly regained the natural color, but were still wanting a full and healthy firmness; a few small points where ulceration had existed were seen, some of which were not yet quite healed. Other abdominal viscera healthy. Gall-bladder moderately full. On examining the brain, its blood-vessels were found considerably congested. No serum in the ventricles or elsewhere. The medullary substance was judged to be more tender and more easily broken down than it is found to be in some other subjects of equal age. This, however, could hardly be regarded as a morbid softening, unless it began to take on such a condition at the time of the first epileptic fit, for, up to that period, the child never showed signs of the least cerebral disturbance whatever. The viscera of the thorax were all natural and healthy. The cavities of the heart were opened and examined. Structure perfect; some small lumps of dark-colored coagula in the left side of the heart. The right ventricle was clear. The right auricle contained a small amount of dark coagulum, and in addition a small body of fleshy color, rather solid, a trifle less than an inch in length and nearly half an inch thick—in shape cylindrical. It was found lying upon the tricuspid valves.

Substances, in appearance like such as have been described in the foregoing cases, are not unfrequently found in the cardiac cavities after death, especially when death follows some acute inflammatory disease. Such, too, are seen commingled with dark coagula in the stillicidious form of uterine hemorrhage, which I have known mistaken for portions of an ovum.

The specimens described were quite firm and compact, more so than is generally found true of such as arise under ordinary circumstances; nevertheless, it is not here claimed that these properties exceeded, in degree, the capacity of pure, unmixed fibrin.

It is regretted that more pains for closer investigation were not taken with the specimens in Cases I. and II. The one in Case III. was submitted to microscopic inspection by competent judges, and pronounced to be fibrin. It is regretted, also, that the attachment in Case I. and II., especially the former, were not more critically examined, so as to ascertain whether or not any bloodvessels passed from the membrane of the cavity into the fibrinous substance, or whether, in fact, it was fastened by anything more than pretty firm adhesion. According to existing evidence, the conclusion may be, that all the bodies described were solid fibrin. Two important questions now arise. Were they of *post* or *ante-mortem* formation? Were they the immediate cause of death in all, or either, of the three cases? My opinion is that the substances were in the heart prior to, and were the cause of, death in *all* of the three cases.

So far as I am informed, nothing has been shown to prove that the fibrin of the blood may not separate, in *some* quantity, from the circulating mass during life; and if it do thus separate in any of the cavities of the heart during the acute stage of an inflammatory disease, may it not remain floating there for a length of time, without perceptibly disturbing the circulation? It can hardly be conceived how any mischief can arise as a consequence, unless such bodies are accidentally thrown into such position as to interfere with the action of the valves. In the foregoing cases, unless the bodies in the heart caused death, the *true* cause remains a problem yet unsolved. That epilepsy in children sometimes becomes fatal, where *post-mortem* appearances hardly show traces of disease sufficient to establish an opinion, beyond doubt, as to the true cause, is probably true, notwithstanding the humiliating nature of the admission. But when dissection discovers such appearances as are described in Cases II. and III., what shall we say? Shall we say that somehow, by some cause inexplicable to us, death came?

A year almost never passes without presenting cases of epilepsy in children, either at the onset or during the acute stage of dysentery; but coming on in the advanced stage of convalescence from disease, and yet depending on the morbid condition alone, is what during my professional life, which is now advanced many degrees beyond its meridian, I have *never* seen.

*Dec. 20th*, 1855.

